# Factors predictive of functional outcomes and quality of life in patients with fragility hip fracture: A retrospective cohort study

**DOI:** 10.1097/MD.0000000000032909

**Published:** 2023-02-17

**Authors:** Mun Jeong Kang, Bo Ryun Kim, Sang Yoon Lee, Jaewon Beom, Jun Hwan Choi, Jae-Young Lim

**Affiliations:** a Department of Physical Medicine and Rehabilitation, Korea University Anam Hospital, Korea University College of Medicine, Seoul, Republic Korea; b Department of Rehabilitation Medicine, Seoul National University Boramae Medical Center, Seoul, Republic of Korea; c Department of Rehabilitation Medicine, Seoul National University Bundang Hospital, Seoul National University College of Medicine, Seongnam-si, Gyeonggi-do, Republic of Korea; d Department of Rehabilitation Medicine, Regional Rheumatoid and Degenerative Arthritis Center, Jeju National University Hospital, Jeju National University College of Medicine, Jeju, Republic of Korea.

**Keywords:** comorbidity, frailty, hip fracture, recovery of function, rehabilitation

## Abstract

To determine the predictors of functional outcomes and quality of life (QoL) of patients who were surgically treated for fragility hip fracture. This was a retrospective cohort study performed in the 3 tertiary rehabilitation facilities. A total of 165 patients who had undergone surgery for fragility hip fracture were followed up to 6 months postoperatively. The factors expected to be related to the functional outcomes and QoL at 6 months post-surgery were as follows: baseline demographics, fracture site, operation type, fall characteristics including fall location and fall direction, comorbidities, and initial functional status. The following were comorbidities: hypertension, diabetes mellitus, dementia, cerebrovascular accident, and osteoporosis. Functional outcome and QoL measures were represented using the Koval grade, functional ambulatory category (FAC), Berg balance scale, 4-m walking speed test, the Korean version of Mini-Mental State Examination, EuroQol 5-dimension (EQ-5D) questionnaire, the Korean version of Modified Barthel Index, and the Korean version of instrumental activities of daily living (K-IADL). For all tests, each patient was assessed immediately after transfer and at 6 months post-surgery. Multivariable regression analyses adjusting for factors mentioned above were as follows. Old age led to a significantly less favorable outcome on FAC and K-IADL at 6 months. Intertrochanteric fracture had a significantly positive impact on Koval at 6 months compared to femur neck and intertrochanteric fractures. Total hip replacement arthroplasty and bipolar hemiarthroplasty had a significantly positive impact on EQ-5D and FAC at 6 months respectively compared to other operation types. Fall characteristics didn’t reveal any significant impact on functional outcomes and QoL. Patients with hypertension and diabetes mellitus had a significantly negative outcome on EQ-5D and K-IADL respectively. Among initial assessments of function and QoL, initial 4-m walking speed test, Korean version of Mini-Mental State Examination, K-IADL, and Korean version of Modified Barthel Index were independent predictors of function and QoL at 6 months. This study confirmed that age, fracture site, operation type, comorbidities, and initial physical and cognitive function significantly influenced recovery of function and QoL at 6 months in patients with fragility hip fractures.

## 1. Introduction

Hip fractures are one of the important causes of disability and death, and their high cost is a significant economic burden throughout the world. Among hip fractures, fragility hip fractures are a global problem as the population ages at a much faster rate than once predicted.^[1]^ Fragility hip fractures are defined as a fracture without identifiable trauma or low-energy trauma, such as a fall from a standing height^[2]^ and most such fractures occur in those in the >65 age group.^[3]^

Although the incidence of hip fractures appears to have declined over the past decade, the prevalence of hip fractures is projected to increase to 550,000 by 2040.^[4]^ As a result, the cost of treating hip fractures will grow to $62 billion by 2040.^[5]^ In 2018, Korea entered an aging society (defined as the elderly population with >14% of the total population) and is expected to become a super-aged society (the elderly population is >20% of the total population) by 2026.^[6]^ Based on these reports, it is expected that the proportion of the elderly population and the number of fragility hip fractures in Korea will show an increasing trend.

After hip fracture, only 40 to 60% of survivors are likely to regain their pre-fracture level of mobility.^[7]^ Although up to 70% can restore a level of independence for basic activities of daily living (ADL), this is variable, and less than half of all people who have experienced a hip fracture can regain their ability to perform instrumental ADL.^[7]^ The 30-day mortality rate was reported to be 3.3 to 17.2% and the 1-year mortality rate was 50%.^[8]^ Mortality rates after hip fracture remains above baseline levels for the first year after fracture as well as for the next 5 years.^[9]^

Therefore, it is essential to establish a specific rehabilitation strategy for maximal functional recovery of patients and for reducing mortality and costs after hip fracture, and research on predictive factors affecting functional outcomes of fragility hip fractures will be helpful for this.

Several studies have been reported on the various factors affecting the postoperative functional outcome in patients with fragility hip fractures. Predictors of poor outcomes identified to date include male patients, people living in support facilities, people with reduced mobility before fractures, and people with depression or dementia.^[10–12]^ Comorbidities (e.g., osteoporosis, diabetes mellitus, cerebrovascular accident, etc.) have also been shown to have a negative impact on functional outcomes.^[13]^

However, among these previous studies, few studies analyzed multiple factors such as fracture site, surgical type, fall location, fall direction, comorbidities, and initial functional status simultaneously and evaluated multiple functional outcome tools at the same time. Because statistical analysis such as multivariable regression results can vary depending on whether each factor is included, a study that includes as many factors as possible at once is necessary.

The aim of this study was to determine predictors of functional outcomes and quality of life (QoL) of patients surgically treated for fragility hip fracture using the aforementioned various factors and assessment tools.

## 2. Methods

### 2.1. Study design

This was a retrospective cohort study performed in 3 tertiary rehabilitation facilities as a part of the clinical trial to investigate the effectiveness of the fragility fracture integrated rehabilitation management program.^[14]^ This study was approved by the Institutional Review Board of Korea University Hospital (IRB no.2021AN0089) and was carried out according to the principles of the Declaration of Helsinki.

### 2.2. Subjects

Between March 2017 and February 2019, patients who had received surgery for a fragility hip fracture and were transferred to the Department of Rehabilitation Medicine and agreed to be enrolled in the study were registered at Seoul National University Bundang Hospital, Chung-Ang University Hospital, and Jeju National University Hospital. All patients were followed up to 6 months postoperatively.

Participants were selected from a larger sample (n = 212) who were enrolled in a multi-site study examining the effectiveness of a home-based fragility fracture integrated rehabilitation management program. Of the full sample, this study enrolled a total of 165 patients (43 males and 122 females; average age 81.1 ± 6.8 years), If they met the following inclusion criteria: age ≥ 65 years; an acute unilateral hip fracture (femoral neck, intertrochanteric, subtrochanteric); and having undergone a successful hip surgery (reduction and internal fixation, bipolar hemiarthroplasty, and total hip replacement arthroplasty). Patients were excluded if they met the following exclusion criteria: a history of a neurodegenerative disease or unstable cardiorespiratory status; having undergone surgery for an infection, arthritis and avascular necrosis; loosening of implants; other kinds of fractures (femoral shaft fractures, acetabular fractures, isolated fractures of the greater or lesser tuberosity, a pathologic fracture caused by a tumor, combined multiple fractures); having undergone revisional surgery; severe cognitive dysfunction (obey command ≤ 1 step); and refusal to participate in the clinical trial.

### 2.3. Outcome measurements

As a part of the clinical trial to investigate the effectiveness of the fragility fracture integrated rehabilitation management program, assessments were selected and conducted to follow up recovery of function and QoL of participants. For all tests, each patient was assessed immediately after transfer and at 6 months post-surgery.

#### 2.3.1. Koval grade.

The Koval grade is a tool that evaluates walking dependency according to 7 grades: independent community ambulator, community ambulator with cane, community ambulator with walker/crutches, independent household ambulator, household ambulator with cane, household ambulator with walker/crutches, and nonfunctional ambulator. Community ambulators were able to walk indoors and outdoors either independently or with assistive devices. Household ambulation was limited to walking indoors either independently or with assistive devices. Nonfunctional ambulators were either bed-bound or limited to bed-to-chair transfers with assistance.^[15]^

#### 2.3.2. Functional ambulatory category (FAC).

The FAC is another tool used to evaluate ambulatory ability by determining how much support the patient needs when walking. It has 6 categories based on the ability to walk at least 10 feet: ranging from (0) inability to walk or walk with the help of ≥2 persons to (5) ability to walk anywhere independently including stairs.^[16]^

#### 2.3.3. Berg balance scale (BBS).

The BBS is used to determine a person’s ability to safely balance. The test consists of 14 predetermined tasks, each of which is scored on a scale from 0 to 4, with a maximum score of 56 indicating good balance. These tasks progress from simple tasks (e.g., sitting to comfortable standing) to more difficult tasks (e.g., tandem standing and single-leg standing).^[17]^

#### 2.3.4. 4-m walking speed test (4MWT).

The 4MWT is a simple test that can measure walking speed. Patients were asked to walk as fast as possible and the time taken to walk 4 m in the middle was measured, excluding the first 1 m for acceleration and the last 1 m for deceleration using a stopwatch.^[18]^

#### 2.3.5. Korean version of Mini-Mental State Examination (K-MMSE).

The K-MMSE is a brief measure of cognitive function, including orientation, memory, language, attention, calculation, praxis, and visuospatial function. It has a maximum score of 30 indicating good cognitive function.^[19]^

#### 2.3.6. EuroQol 5-dimension questionnaire (EQ-5D).

The EQ-5D questionnaire is widely used to evaluate self-reported QoL and general health status. It has 5 dimensions: mobility, self-care, usual activities, pain/discomfort, and anxiety/depression. Each dimension has 1 question with 3 severity levels (no problems, some or moderate problems, and extreme problems).^[20]^ The scores were converted using utility weights obtained from general Korean population and ranged from −1 to 1, with higher scores for higher health-related QoL.^[21]^

#### 2.3.7. Korean version of modified Barthel index (K-MBI).

The K-MBI is a tool for assessing individual performance. It measures 10 items of activities of daily life and items consist of 2 groups: self-care (feeding, grooming, bathing, dressing, bowel and bladder care, and toilet use) and mobility (ambulation, transfers, and stair climbing). Scores range from 0 (completely dependent) to 100 (completely independent).^[22]^

#### 2.3.8. Korean version of instrumental ADL (K-IADL).

The K-IADL is used to evaluate the ability to perform instrumental ADLs that allow an individual to live independently in a community. It has 10 items: decorating, housework, preparing meals, laundry, going out for a short distance, using transportation, shopping, handling money, using a telephone, and taking medicine. Three items use a 3-point scoring system and 7 items use a 4-point scoring system. The average score is obtained by dividing the total score by the total number of items. The higher the score, the lower the function.^[23]^

### 2.4. Statistical analysis

All statistical analyses were performed using Statistical Package for the Social Sciences version 24.0 (IBM, Armonk, NY). Baseline demographics were analyzed using descriptive statistics. A paired *t* test was used to compare differences in physical function and QoL immediately after transfer and 6 months post-surgery. Multivariable linear regression analysis, adjusted for age, sex, hospital, fracture site, operation type, fall location, fall direction, comorbidities, initial variables of the functional outcomes, and QoL was used to identify variables that had a significant effect on physical function and QoL 6 months post-surgery. The *P* value < .05 was indicating statistical significance.

## 3. Result

### 3.1. Demographic results

The demographic characteristics of the patients are listed in Table 1. A total of 165 patients were enrolled in the study. The mean age of all patients was 81.1 ± 6.8 years. Forty-three patients were male and 122 were female. The patients were classified into several groups based on each of the following characteristics: fracture site (71 femur neck; 86 intertrochanteric; 8 subtrochanteric); operation type (76 bipolar hemiarthroplasty; 10 total hip replacement arthroplasty; 79 reduction and internal fixation); fall location (41 indoor; 124 outdoor) and fall direction (28 anterior; 74 sideway, 43 posterior). The prevalence of hypertension, diabetes mellitus, dementia, cerebrovascular accident, and osteoporosis was 67.9% (112), 35.8% (59), 7.3% (12), 15.8% (26), and 73.3% (121), respectively.

**Table 1:** Demographics of the subjects (N = 165).

Variables	Values
Age (yr)	81.1 ± 6.8
Sex, males/females (number)	43 (26.1)/ 122 (73.9)
Height (cm)	155.9 ± 8.7
Weight (kg)	54.6 ± 9.6
Hospital (number)	
A	61. (37.0)
B	57 (34.5)
C	47 (28.5)
Fracture site (number)	
Femur neck	71 (43.0)
Intertrochanteric	86 (52.1)
Subtrochanteric	8 (4.8)
Operation type (number)	
Bipolar hemiarthroplasty	76 (46.1)
Total hip replacement arthroplasty	10 (6.1)
Reduction and internal fixation	79 (47.9)
Fall location (number)	
Indoor	41 (24.8)
Outdoor	124 (75.2)
Fall direction (number)	
Anterior	28 (17.0)
Sideway	74 (44.8)
Posterior	43 (38.2)
Time from surgery to RM transfer (d)	7.4 ± 2.9
Hospitalization period at RM (d)	13.7 ± 4.7
Comorbidities (number)	
Hypertension	112 (67.9)
Diabetes mellitus	59 (35.8)
Dementia	12 (7.3)
Cerebrovascular accident	26 (15.8)
Osteoporosis	121 (73.3)

Values represent mean ± standard deviation or number (%) of cases.

### 3.2. Evaluation of function and QoL immediately after transfer and 6 months post surgery

Table 2 shows the evaluation of function and QoL for all patients immediately after transfer and 6 months post-surgery. The scores of all functional outcomes and QoL were improved significantly 6 months post-surgery compared with immediately after the transfer (*P* < .05).

**Table 2:** The evaluation of physical and cognitive function and quality of life immediately after transfer and at 6 months post-surgery (N = 165).

Variable	Values		
	Immediately after transfer	6 months post surgery	*P* value
Koval grade	6.64 ± 0.51	3.16 ± 1.92	<.001
FAC	0.82 ± 1.07	3.64 ± 1.25	<.001
BBS	12.45 ± 12.72	37.86 ± 16.36	<.001
4MWT (s)	26.07 ± 15.76	10.09 ± 9.21	<.001
K-MMSE	19.86 ± 6.55	20.14 ± 9.18	.044
EQ-5D	0.38 ± 0.17	0.69 ± 0.17	<.001
K-MBI	43.85 ± 17.40	78.63 ± 28.49	<.001
K-IADL	28.32 ± 3.47	21.05 ± 6.11	<.001

Values represent mean ± standard deviation.

4MWT = 4-m walking speed test, BBS = Berg balance scale, EQ-5D = EuroQol 5-dimension, FAC = functional ambulatory category, K-IADL = Korean version of instrumental activities of daily living, K-MBI = Korean version of Modified Barthel Index, K-MMSE = Korean version of Mini-Mental State Examination.

### 3.3. Predictors of functional outcome 6 months after fragility hip fracture surgeries

The results of the multiple regression analysis are summarized in Table 3. Multivariable regression analyses adjusting for age, sex, hospital, fracture site, operation type, fall location, fall direction, comorbidities, and initial variables of the functional outcomes and QoL were as follows.

**Table 3:** Predictors of functional outcome and quality of life at 6 months after fragility hip fracture surgery.

Functional outcome and quality of life/Independent predictor	Standardized (*ß*)	*P* value	Adjusted *R*^2^
Koval grade			0.350
Fracture site, intertrochanteric	0.324	.014	
Initial 4MWT	0.304	.022	
Initial K-IADL	0.322	.011	
FAC			0.310
Age	−0.477	<.001	
Operation type, bipolar hemiarthroplasty	0.284	.033	
Diabetes mellitus	−0.366	.006	
BBS			0.203
Initial 4MWT	−0.366	.015	
Initial K-MMSE	0.340	.023	
4MWT			0.274
Initial 4MWT	0.385	.012	
Initial K-IADL	0.441	.004	
K-MMSE			0.514
Sex	0.269	.028	
Initial 4MWT	−0.247	.033	
Initial K-MMSE	0.744	<.001	
EQ-5D			0.148
Operation type, total hip replacement arthroplasty	0.300	.029	
Hypertension	−0.326	.018	
K-MBI			0.204
Initial 4MWT	−0.338	.020	
Initial K-MMSE	0.355	.015	
K-IADL			0.410
Age	0.286	.017	
Initial K-MBI	−0.438	<.001	
Diabetes mellitus	0.314	.008	

The multivariable regression analyses adjusting for age, sex, hospital, fracture type, operation type, fall location, fall direction, comorbidities, initial variable of the functional outcomes, and quality of life.

4MWT = 4-m walking speed test, BBS = Berg balance scale, EQ-5D = EuroQol 5-dimension, FAC = functional ambulatory category, K-IADL = Korean version of instrumental activities of daily living, K-MBI = Korean version of Modified Barthel Index, K-MMSE = Korean version of Mini-Mental State Examination.

Old age led to significantly less favorable outcomes on FAC and K-IADL at 6 months.

Intertrochanteric fracture had a significantly positive impact on Koval at 6 months compared to femur neck and intertrochanteric fractures.

Total hip replacement arthroplasty had a significantly positive impact on EQ-5D at 6 months compared to bipolar hemiarthroplasty (BPH) and reduction and internal fixation. BPH had a significantly positive outcome on FAC at 6 months compared to other operation types.

Patients with hypertension had a significantly negative outcome on EQ-5D and patients with diabetes mellitus had a significantly less favorable outcome on K-IADL.

Among initial functional assessments, initial 4MWT was an independent predictor of Koval, BBS, 4MWT, K-MMSE, and K-MBI at 6 months. Initial K-MMSE was significantly associated with BBS, K-MMSE, and K-MBI at 6 months. Initial K-IADL was an independent predictor of Koval and 4MWT at 6 months and initial K-MBI was an independent predictor of K-IADL at 6 months.

Fall characteristics didn’t reveal any significant impact on functional outcomes and QoL.

## 4. Discussion

This study demonstrates that various factors such as fracture site, surgical type, comorbidities, and pre-fracture functional status significantly influence physical function outcomes and QoL 6 months after fragility hip fracture surgeries, thus, providing an integrated, rather than fragmentary, view of the patient’s recovery after hip fracture surgery.

First of all, our results are concordant with previous studies showing the association between age and gait recovery in patients with fragility hip fractures. In this study, FAC scored lower in older patients at 6 months post-surgery, meaning that older patients had less gait recovery than younger patients. This finding is similar to that of Takahashi et al^[24]^ who used the same scale of FAC. They concluded that old age negatively affects ambulatory functional outcomes in patients with hip fractures. Wong et al^[25]^ also argued that the risk of ambulation deterioration increased every 10 years of age.

In addition, our study showed that K-IADL scored higher in older patients at 6 months post-surgery, meaning that older patients had less independence in daily life than younger patients. Many studies have shown that older patients had a poor recovery to pre-fracture ADL.^[26–28]^ Mayoral et al^[26]^ argued that comorbidities, cognitive status, and previous physical state affect poor recovery of older patients. Considering the above hypotheses and that there was no significant difference in MMSE scores by age in our study, the negative effect of age on the FAC and K-IADL scores in our study can be considered to be due to the combination of physical state and comorbidities.

There have been relatively many studies on mortality and ambulatory function after surgery according to fracture type.^[24,29–36]^ There was a study that showed no difference in mortality or function according to fracture type,^[36]^ and there were rare reports that intertrochanteric fracture had worse function,^[29,35]^ but overall, intertrochanteric fracture reported worse initial function,^[24,30]^ mortality,^[31,32]^ and functional outcomes.^[24,30,33,34]^

Consistent with past reports, intertrochanteric fracture had a significantly higher Koval grade in this study and it indicates that patients with intertrochanteric fracture have significantly poor recovery of walking ability. A possible explanation of this finding may be the biologically older age of patients with intertrochanteric fractures. Lawton et al^[37]^ reported that patients with intertrochanteric fractures had poorer pre-fracture ambulatory function and more associated medical conditions, such as anemia, that affected fracture management than patients with femoral neck fractures. Similarly, Jarnlo et al^[38]^ also reported that patients with intertrochanteric fractures seemed to be less active than patients with femoral neck fractures. Another possible explanation may be pain, reduced knee-extension strength, and larger edema in the thigh with intertrochanteric fracture.^[39,40]^

There was a significant difference in functional outcome and QoL according to operation type in our study. Several studies have investigated the relationship between postoperative functional outcomes and surgical type, and the results have been varied.^[36,41–46]^ There were reports that there was no difference in functional outcome depending on the surgical method,^[36]^ or that internal fixation produced better results,^[41]^ but in most cases, arthroplasty showed better results than internal fixation and total hip replacement showed better results than BPH among arthroplasty.

Chammout et al^[45]^ who compared the results of total hip replacement with those of internal fixation over a long-term follow-up period of 17 years concluded that total hip replacement provided better hip function and significantly fewer reoperations compared with internal fixation without increasing mortality. Alexiou et al^[46]^ assessed 49 randomized controlled trials or prospective cohort studies reporting the QoL and psychological outcomes and demonstrated that arthroplasty had better functional outcomes and QoL than internal fixation.

In our study, total hip replacement arthroplasty and BPH showed significantly higher EQ-5D and FAC, respectively. In other words, arthroplasty showed better results compared to internal fixation in QoL and ambulatory function, so the results were consistent with the literature.

In terms of the relationship between hypertension and fragility hip fractures, EQ-5D related to QoL scored lower in patients with hypertension in our study. The association between hypertension and QoL has been proven through many studies.^[47–49]^ Bardage et al^[47]^ demonstrated that hypertensive patients had significantly lower QoL, with lower health-related QoL and 36-item short-form questionnaire scores. Previous studies explained that hypertensive individuals have reported lower social and psychological functioning^[50,51]^ and have been associated with symptoms^[52,53]^ and/or side effects of antihypertensive medication^[54,55]^ such as headache, dizziness, depression, anxiety, and tiredness.

A link between hypertension and frailty has also been established in many studies.^[56–58]^ Kang et al^[56]^ argued that uncontrolled hypertension can cause serious cardiovascular events and hypertension is related to future ADL/IADL limitation or disability. In this regard, in patients with hip fractures, it can be considered that hypertension-related deterioration of social and psychological function, symptoms, and frailty will cause a decrease in QoL.

With respect to the relationship between diabetes mellitus and fragility hip fractures, K-IADL scored higher in patients with diabetes mellitus in our study. It is well known that diabetes is a risk factor for fragility hip fractures^[59]^ and several studies on the effects of diabetes mellitus on functional outcomes and mortality post-surgery in patients with fragility hip fractures showed a negative effect.^[60–62]^

Yoon et al^[60]^ found that diabetes negatively affected a variety of functional outcomes, including not only K-IADL and also the FAC, EQ-5D, K-FRAIL, and geriatric depression scale related to depression. Lieberman et al^[61]^ concluded that the rehabilitation outcome of diabetic patients was significantly worse than that of non-diabetic patients among older patients with hip fractures using the FIM scale. They explained diabetic patients have an increased risk for medical complications such as cerebrovascular and cardiovascular events and surgical site infection. Rutenberg et al^[62]^ demonstrated higher mortality, more medical complications, and longer rehabilitation duration in diabetic patients with fragility hip fractures.

On the other hand, Tian et al^[63]^ argued that diabetes does not affect post-fragility hip fracture functional outcomes and mortality. However, they also reported a higher incidence of postoperative complications, such as urinary tract infections and deep vein thrombosis in patients with diabetes. Therefore, decreased ability of diabetic patients to perform daily activities can be thought to be primarily due to medical complications.

Initial functional status is one of the most well-known predictive factors of functional outcome after hip fracture surgery. Many studies have already reported associations between various initial functional statuses, such as cognition,^[64–74]^ gait,^[24,64,68,71,75–77]^ and ADL,^[78–82]^ and postoperative functional outcomes using various evaluation tools.

It has been reported that poor cognitive function significantly adversely affects the functional recovery of gait and ADL after hip fracture surgery.^[64–74]^ Liang et al^[72]^ demonstrated that patients who had cognitive impairment showed significantly less improvement in ambulatory status than reference patients and significantly lower MBI scores after hip fracture rehabilitation. Söderqvist et al^[74]^ found that cognitive dysfunction of patients with hip fractures effectively predicted their outcome with regard to the ability to walk and perform ADL.

It has also been studied that the initial ambulatory state and ADL state significantly affect gait and ADL function recovery after hip fracture surgery. Several studies reported that patients with better scores on the Timed Up and Go Test,^[77]^ BBS,^[71]^ and FAC^[24]^ showed significantly good recovery of ambulatory function after hip fracture surgery. Other studies found that patients with good ambulatory ability after hip fracture surgery have also been more likely to regain their pre-fracture independent living status. Initial ADL function was found that independent predictor of ADL and QoL after hip fracture surgery. Ishidou et al^[79]^ showed that an increased risk of BI deterioration was associated with worse BI at discharge and Chang et al^[82]^ reported that poor baseline ADL could predict poor QoL 6 months after hip fracture surgery.

Based on these results, it can be seen that the functional state does not affect cognition, gait, and ADL separately, but complexly influences each other. Thus, our findings support previous studies. It was found that cognition, gait, and ADL influence each other in this study. Among them, initial 4MWT showed a significant difference in the most functional outcomes (Koval, BBS, 4 MWT, K MMSE, and K MBI) and MMSE also showed a significant difference in BBS, MMSE, and MBI. Therefore, evaluating cognition, gait speed, and ADL will be a simple and useful method for predicting prognosis and preparing for rehabilitation strategy in clinical practice.

Previous studies demonstrated that there is a direction of loading in which a hip fracture easily occurs^[83,84]^ and several studies have been conducted on the fall characteristics as a risk factor for hip fracture.^[85,86]^ Hwang et al^[86]^ reported that sideway falls and backward falls were associated with 12.8- to 15.2- and 9.86- to 10.8-fold increased risks of hip fractures, respectively. Cumming et al^[87]^ hypothesized that a sideways fall would likely absorb the impact directly because there is less soft tissue such as skin, fat, and muscle.

However, no study was conducted on whether the fall characteristics such as direction and location affect the functional outcomes and QoL after hip fracture surgery. In this study, fall direction didn’t reveal any significant impact on functional outcomes. This suggests that the fall direction has a significant effect on the direction and value of the load and therefore affects the risk of hip fracture, but once a load above the threshold sufficient to induce a hip fracture is applied, the fall direction does not significantly affect the functional outcome.

The fall location showed different indoor and outdoor ratios in various studies,^[87,88]^ and to our knowledge, it was not reported as an independent risk factor for hip fracture. In our study, outdoor was about 3 times indoor, but it did not have a significant effect on functional outcomes. However, further studies are needed to determine the relationship between fall characteristics and postoperative functional outcomes and QoL in patients with fragility hip fractures. It can be helpful to study the relationship between the fall direction and fall location as well as the situation or behavior at the time of the fall and functional outcomes.

### 4.1. Limitation

Our study has several limitations. First, because the study was conducted on Korean patients who received rehabilitation treatment at a tertiary hospital, the study population may not represent the general community-dwelling older adults who have suffered from fragility hip fractures. Second, the pre-fracture functional status was not evaluated in this study. Pre-fracture functional status is one of the best-known predictive factors of functional outcome after hip fracture surgery.^[26,89–92]^ Since preoperative functional status is likely to be poor in patients with older age, comorbidities, and intertrochanteric fracture, further studies are needed to evaluate the effect of this on postoperative functional outcomes. Third, among the numerous comorbidities, only comorbidities with a relatively high frequency could be studied. Therefore, larger studies are needed to evaluate the effects of these factors on the functional outcome post-fragility hip fracture surgeries.

Despite the limitations, this study has several strengths. First, this study has identified and clarified which factors can affect the postoperative functional outcomes. Therefore, clinicians can focus on intervening in certain factors such as comorbidities as well as impaired initial function that should be intensively controlled and providing individualized rehabilitation strategies. Second, we investigated the effects of various factors and used a lot of functional outcome tools at once. This provided an integrated, rather than fragmentary, view of the patient’s recovery after hip fracture surgery.

## 5. Conclusion

This study demonstrated that age, fracture site, operation type, comorbidities, initial cognition, gait speed, and ADL significantly influenced the recovery of function and QoL at 6 months in patients with fragility hip fractures. Old age, intertrochanteric fracture, hypertension, diabetes mellitus, poor cognition, lower gait speed, and poor ADL had a significantly negative outcome and total hip replacement arthroplasty and BPH had a significantly positive outcome. However, fall characteristics such as fall location and fall direction didn’t reveal any significant impact on functional outcomes and QoL in this study.

Therefore, when establishing an initial rehabilitation plan for patients with surgically treated for fragility hip fracture, it is necessary to recognize the effects of the aforementioned factors and to establish individualized goals and treatment programs.

## Acknowledgments

We would like to thank Korea University Medical Library (https://medlib.korea.ac.kr/) for the English language editing.

## Author contributions

**Conceptualization:** Mun Jeong Kang, Bo Ryun Kim.

**Data curation:** Sang Yoon Lee, Jaewon Beom, Jun Hwan Choi, Jae-Young Lim.

**Writing – original draft:** Mun Jeong Kang.

**Writing – review & editing:** Bo Ryun Kim.
